# Push-Out Bond Strength Assessment of Different Post Systems at Different Radicular Levels of Endodontically Treated Teeth

**DOI:** 10.3390/ma15155134

**Published:** 2022-07-24

**Authors:** Valérie Kanzler Abdel Raouf, Julia Jockusch, Nadin Al-Haj Husain, Nataliya Dydyk, Mutlu Özcan

**Affiliations:** 1 Division of Dental Biomaterials, Clinic of Reconstructive Dentistry, Center of Dental Medicine, University of Zurich, 8032 Zurich, Switzerland; valerie.kanzler@uzh.ch (V.K.A.R.); nadin.al-haj-husain@unibe.ch (N.A.-H.H.); ndydyk@gmail.com (N.D.); 2 University Research Priority Program Dynamics of Healthy Aging“, University of Zurich, 8050 Zurich, Switzerland; julia.jockusch@uzh.ch; 3 Department of Reconstructive Dentistry and Gerodontology, School of Dental Medicine, University of Bern, 3010 Bern, Switzerland; 4 Department of Prosthetic Dentistry, Danylo Halytskyy Lviv National Medical University, 79010 Lviv, Ukraine

**Keywords:** adhesion, bond strength, dental materials, endodontics, fiber-reinforced-composite posts, intraradicular posts, prosthetic dentistry, push-out test

## Abstract

This study assessed the bond strength of prefabricated post systems at different root levels of endodontically treated teeth. One-rooted human premolars (N = 70; n = 10) were cut to 2 mm above the cement-enamel junction. Root canals were treated and randomly assigned to one of the seven post systems: T: Titanium (Mooser), ZrO: Zirconia (Cosmopost), G: Fiber (FRC Postec Plus), E1: Fiber (Direct) (Everstick post), E2: Fiber (Indirect) (Everstick post), PP: Fiber (PinPost), and LP: Injectable Resin/Fiber composite (EverX Posterior). All posts were luted using a resin cement (Variolink II), and the roots were sectioned at the coronal, middle, and apical root levels. Push-out tests were performed in the Universal Testing Machine (0.5 mm/min). Data (MPa) were analyzed using two-way ANOVA and Tukey’s tests (α = 0.05). The results showed that the bond strength (mean ± SD) of E2 posts were highest (5.3 ± 2.7) followed by PP (4.1 ± 2.0); G (4.0 ± 1.6); LP (2.6 ± 1.9): T (2.2 ± 1.5) and ZrO (1.9 ± 1.0) posts systems. No significant differences were found in bond strength of all post systems. The bond strength in the coronal root level was the highest with 3.6 ± 2.2 MPa. The bond strength of FRC post systems was significantly higher than those of rigid posts of titanium or ZrO_2_. Bond strength results were the highest in the coronal root level for all tested post systems but did not differ significantly from the other two root levels.

## 1. Introduction

The main clinical goal of using a post system is to provide additional support and retention to the coronal restoration in endodontically treated teeth with compromised crown structure [1]. For that purpose, different treatment modalities and materials have emerged to maximize the clinical outcomes of biomechanical stability, esthetics and longevity [2].

Multiple parameters concerning the intraradicular post influence the success of restored endodontically treated teeth. For instance, the adhesion of the post to the intraradicular dentin and its retention within the radicular structure has an impact on restorative complex durability. Further factors include the amount of remaining tooth structure and post, as well as core and cement material properties [3,4,5].

Cast and prefabricated posts, with various geometries and sizes, have been in clinical use for decades [6,7]. With the higher demand in esthetic dentistry, tooth-colored posts have been increasingly developed using various materials [8,9]. The use of metallic posts resulted in root discoloration (blue-gray) that could show through the overlaying soft tissue and consequently impair an ideal esthetic outcome. Similarly, a metallic core could influence the optical properties of the ceramic restorations, which could make a metal post and core a challenging and unpredictable esthetic treatment option [9,10].

Fiber-reinforced composite (FRC) and zirconia posts have been offering tooth-colored post solutions. FRC have demonstrated a considerable success over a long-term clinical follow up period [11,12]. FRC was introduced in early 1990 as carbon fibers post system, and since then, many other FRC post composites have been developed and used clinically. Concomitantly, there has been a remarkable advancement in adhesive dentistry, which enhanced the effectiveness of bondable FRC posts and their core complexes. The clinical performance of FRC has mainly been attributed to its bonding ability and biomimetic behavior. Numerous studies have indicated that the most prevalent failure pattern in fiber post-based restorations is post debonding [13,14].

The integrated adhesion of the post to the core and to dentin plays an important role in the overall clinical performance of such restorations. Despite the remarkable advancement in bonding techniques and materials, attaining a long-term constant and predictable bonding to intraradicular dentin remains a clinical challenge [4,15]. Therefore, it has been of interest to investigate the type of post and adhesive system that could meet the best biomechanical qualities, esthetics and long-term reliability [3,16].

Rigid posts, metallic and zirconia post systems, have very high elastic moduli compared to dentine, which could induce the concentrated internal stress to cause root fracture. On the other hand, semi-rigid post systems, FRC, have demonstrated more disperse force distribution and reduced risk of root fracture owing to their dentine-similar elastic moduli [12,15,17]. FRC posts are manufactured by having different fibers of carbon, quartz or glass embedded into different matrices of epoxy and methacrylate resin. Fibers within its matrix are in a longitudinal direction parallel oriented to the long axis of post. The characteristics of the fibers differ in term of diameter (6–15 μm) and density (25–35 fiber/mm^2^) within a post [2,8].

One of the shortcomings of using a prefabricated post system is the need to shape the canal to accommodate the post of choice, which is an additional removal of tooth structure. Moreover, root canal three-dimensional shape (e.g., curved canal) is a main factor in post selection. Recent advances have provided options of prefabricated, unpolymerized posts to fit the canals form or in an injectable fashion to eliminate the need for additional tooth structure removal. In such modalities, post-polymerization adhesive and biomechanical properties are the main influential factors of question, where matching drills is irrelevant to their fit [9,18,19,20,21,22]. Great research interest for selection of the optimal post type that would exhibit the highest bond strength in a root canal has been advocated, as numerous clinical studies have shown that the most frequent types of failure associated with post restorations of endodontically treated teeth were debonding and loss of retention [4,13,14]. As disclosed in earlier studies the unfavorable C-factor, the incomplete polymerization and the induced shrinking stress decreases the bond strength to intraradicular dentin where an increasing C-factor may lead to debonding at the post-dentin interface [23]. Therefore, the aim of this study was to investigate the push-out bond strength of different prefabricated post systems, at different root levels of endodontically treated teeth and provide clinical recommendations.

## 2. Material and Methods

### 2.1. Specimen Preparation

Single-rooted human premolars (N = 70; n = 10) were used for the preparation of the specimens. The sample size was determined according a previously published study [5]. All teeth used in the present study were extracted for reasons unrelated to this project. Written informed consent for research purpose of the extracted teeth was obtained by all donors prior to extraction according to the directives set by the National Federal Council. Ethical guidelines were strictly followed, and irreversible anonymization was performed in accordance with State and Federal Law [23,24,25]. After extraction, teeth were stored in distilled water at 5 °C for a maximum of three weeks until use [26]. The inclusion criteria for the selection of the teeth were as follows:

- straight roots
- round root canal form
- absence of crown/root decay, cracks, and previous endodontic treatment
- root length of at least 16 mm.

All specimens were prepared by one investigator. After the removal of the clinical crowns up to 2 mm above the cement-enamel junction a root canal treatment was performed. Root canals were prepared by using the Protaper Profile Orifice Shapers System (Dentsply Maillefer, Tulsa, UK) with an additional root canal preparation lubrication (Premier Dental Products, Plymouth, PA, USA), until the working length (1 mm above the apical foramen) was reached with the file F3. Additionally, the root canal was irrigated with 5 mL of 2.5% sodium hypochlorite for 5 min. The root canals then were filled using the lateral condensation technique with gutta percha cones and AH Plus resin sealer (Dentsply, York, PA, USA). After the removal of the coronal gutta-percha from the root walls, all specimens were stored in physiological saline solution at 37 °C for 7 days.

All premolars (n = 70) were randomly assigned to one of seven post systems used in this study (Table 1).

The preparation of the root canals was carried out for each post system according to the manufacturer’s instructions. The root canal filling was removed up to 4 mm apical length, to allow a post length of at least 12 mm. The post length was controlled throughout the experimental procedures. The canal was thereafter prepared to the required depth using the corresponding post size instrument and reamer at 1000–5000 rpm. Each bur was used a maximum of five times. Afterwards, the root canals were rinsed with water. Excess moisture was removed using paper points. All posts were cemented using conventional resin cement (Variolink II, Ivoclar Vivadent, Schaan, Lichtenstein) according to the manufacturer’s instructions. The dentin surface was etched using 37% phosphoric acid for 10–15 s, rinsed for 5 s and air-dried. The dentin surface was conditioned using an adhesive system (Syntac Classic, Ivoclar Vivadent, Schaan, Lichtenstein), including conditioning for 15 s with the Syntac primer, air-thinning, application of Syntac adhesive for 10 s, air-thinning and Heliobond application and light-curing for 10 s. Light-curing procedures for the post if necessary were carried out using at an intensity of 1′200 mW/cm^2^ and suitable for the wavelength (385–515 nm) (Bluephase, Ivoclar Vivadent, Schaan, Liechtenstein) at 2 mm occlusal distance for 60 s. The roots were sliced into three sections by using a low-speed diamond blade (Isomet, 1000, Buehler Ltd., Lake Bluff, IL, USA) under water-cooling after they were embedded in auto-polymerized acrylic resin blocks (Orthoresin, Dentsply/DeTrey, Konstanz, Germany). The thickness of each slice was measured using a digital caliper (0.01 mm accuracy; Mitutoyo, Tokyo, Japan). The resulting three slices represented different root levels: the coronal level, the middle level, and the apical root level.

### 2.2. Measuring Method

Push-out bond strength was measured by using a cylindrical plunger mounted on the Universal Testing Machine (Model LRX-plus, Lloyd instruments Ltd., Fareham, UK). Compressive load was applied at a crosshead speed of 0.5 mm/min until the post segment was dislodged from the root to the apical aspect in the apical-coronal direction. The plunger tip size was selected and positioned to contact only the post, without stressing the surrounding root canal walls.

The initial bond strength (in MPa) was calculated:

$$Bond\;strength\;\left(\mathrm{MPa}\right)=\frac{\mathrm{maximum}\;\mathrm{load}\;\left(N\right)\;}{\mathrm{area}\;\mathrm{of}\;\mathrm{adhesion}\;\mathrm{surface}\;\left({\mathrm{mm}}^{2}\right)}$$

The adhesion area of each section is the area of the lateral surface of a cone. It was calculated as follows:

SI=π (r+R)a

π *= 3.14, R—coronal radius, r—apical radius, a—apothem.*

The apothem was computed using the formula:

$$a={\left[h^{2}+{\left(R-r\right)}^{2}\right]}^{1/2}$$

*h*—thickness of the slice.

### 2.3. Statistical Analysis

All statistical analyses were carried out using SPSS 15.0 software (IBM, Somers, NY, USA). Mean values and standard deviation (SD) were calculated. After normality testing using a Kolmogorov–Smirnov test, two-way ANOVA was run to analyze the means of each post group. The Tukey’s post hoc test was used to make multiple comparisons between all groups. The significance level was set at α < 0.05.

## 3. Results

Considering the different post systems, the results showed that the bond strength of E2 posts were highest (mean ± SD: 5.3 ± 2.7) followed by PP (4.1 ± 2.0) and G (4.0 ± 1.6) posts (Table 2). E2 post showed significantly higher mean bond strength values than all other post systems compared to. T and ZrO post showed significantly lower mean bond strength values than G, E1, E2, and PP post systems. G and E1 posts each showed significantly higher mean bond strength values compared to T, ZrO, and LP posts, while they showed significant lower values compared to E2 posts. PP post mean bond strength values were significant higher compared to T, ZrO, and LP posts but lower compared to E2 posts. Furthermore, LP post mean bond strength values were significantly lower compared to G, E1, E2, and PP post systems.

With regard to the root level, no significant differences in bond strength of all post systems were found. The observed bond strength in the coronal root level was highest with 3.6 ± 2.2, but did not differ from the other two root levels (Table 3).

Only PP posts showed the highest push-out bond strength at the apical root level (mean ± SD: 4.7± 2.6), whereas all other post systems showed the highest bond strength at the coronal (mean ± SD: ZrO 2.0 ± 1.1; E1 4.2 ± 2.3; LP 3.9 ± 2.1) or mid-root level (T 2.7 ± 2.1; G: 4.4 ± 1.4.; E2 5.3 ± 2.9) (Table 4).

The significance values of the mean bond strength values of all post systems (T, ZrO, G, E1, E2, PP, and LP) at all three root levels (coronal, middle and apical) have been compared to each other (Table 4).

### 3.1. Bond Strength at Coronal Root Level

Regarding the mean push-out bond strength at the coronal root level, significant differences have been found for the T post and the E1, E2, and PP post systems, thereby T post showed the lowest bond strength. Additionally, ZrO posts showed significant differences in bond strength at the coronal root level compared to E1, E2, and PP, thereby ZrO post showed the lowest bond strength. E1, E2, and PP post each showed significant higher bond strength at the coronal level compared to T and ZrO posts. No differences have been found for G and LP post compared to all other post system at the coronal level (Table 4).

### 3.2. Bond Strength at Middle Root Level

At the middle root level, T post showed a significant lower bond strength than E2 post. In addition, for ZrO posts significant lower bonding strength values at the middle root level have been observed compared to G and E2 post. G posts showed a significant higher bond strength at middle root levels compared to ZrO, and LP. The same applies to E2 compared to T, ZrO, E1, and LP posts. Significant lower mean bonding strength values at the middle root level were measured for LP post compared to G and E2 posts, and E1 compared to E2 post (Table 4).

### 3.3. Bond Strength at Apical Root Level

At the apical root level T and ZrO post showed significant lower mean bond strength values compared to E2 and PP posts, while LP posts showed significant lower values compared to G, E1, E2, and PP posts. Significant higher mean bond strength values were recorded for G and E1 posts compared to LP post, and E2 and PP posts each compared to T, ZrO, and LP posts (Table 4).

The failure modes were exclusively mixed types of failures including partial detachment of the resin cement from the root surface and the intra-radicular post from the cement surface.

## 4. Discussion

In the present study, the push-out bonding test has been applied for measuring the bond strengths of posts to intra-radicular dentin. The push-out bonding test has been considered as a reliable method that provides a better estimation of the bond strength of posts than does the conventional shear test. It has been applied in several recent studies assessing the influence of a range of factors on bond strength of different types of posts and luting agents [27,28,29]. One of the major advantages of the push-out test, is that the fracture occurs parallel to the dentin–adhesive interface, which makes it a true shear test [28]. Retention of adhesively luted fiber-reinforced posts relies on the strength of the bonding interface between dentinal root canal wall on one hand and the post surface on the other. It is important that the bond strength is sufficiently strong to withstand stresses during functional loading. The recent research focusing on fiber-reinforced posts and their comparison to other types of posts concluded the tendency of increasing use of fiber posts as an alternative to metal posts in the restoration of endodontically treated teeth [3,4,5]. The use of FRC posts is considered as a minimal invasive procedure, since they could be adhesively bonded to the root canal dentin, do not require extensive root canal preparation, preserve the root structure and cause less root fracture [6]. Moreover, translucent FRC posts are also considered optically more favorable in aesthetically demanding regions compared to metal posts [11]. Finally, it has been reported that FRC post systems are clinically superior due to their more favorable failure modes compared to metal post systems [16,17].

Based on the results of this study, the type of post material significantly affected the push-out bond strength results. Among all post types FRC posts demonstrated overall superiority in adhesion to root canal walls compared to titanium T and Zirconium oxide post ZrO. Due to impregnation with a resin monomer network, interpenetrating the polymer network, a copolymerization high bond strength could be attributed to this reason. Yet, the difference in terms of bond strength was not above 3.4 MPa. It was shown that the bond strength of E2 posts (FRC post) was higher than all other tested post systems followed by PP and G posts. The highest bond strength values of FRC posts in combination with other favorable in vitro physical and mechanical properties that have been demonstrated in recent studies supports their clinical use [8]. Especially in teeth with extensive coronal destruction the clinical outcome advantages of fiber-reinforced composite post have been reported [8]. Bond strength results that influence a performance of FRP in restorations of endodontically treated teeth provide valuable information to predict the clinical outcome and expect decreasing of debonding frequency due to appropriate selection of post type. Nevertheless, the in-vivo survival of FRC posts and debonding occurrence must be further investigated.

When using FRC posts, it must be taken into consideration that the bond strength values are dependent from the type of post used–prefabricated or individually formed [30]. Prefabricated FRC posts seem to have lower bond strength than individually formed FRC post [29], most likely due to the lack of radicals of the prefabricated FRC opposed to the reactivated posts with adhesive resin.

Rigid post systems (titanium, ZrO_2_) revealed the lowest bond strength results. These finding are in agreement with other studies demonstrating lack of adhesion between the resin cement and the post surface for T and ZrO, as physical-chemical bond is inferior. In contrast, Perdigão. et al. [24] did not observe significant differences among different types of fiber posts. However, bond strength to fiber posts was found to be superior compared to bond strength to zirconia posts [24]. Additionally, a study from Al-Tayyan et al. [18] showed that the mean axial resistance forces of flexible fiber-bundle dowel system and that of rigid prefabricated fiber dowel system do not differ [18]. In contrast, Parčina and Amižić et al. [30] demonstrated a significant difference in the bond strength between the root levels wherein the apical root level was outnumbered [29].

A study by Alnaqbi et al. [31] reported differences in the push-out bond strength dependent of the type of post-matrix system used. They demonstrated-in contrast to this study that IPN Everstick posts revealed the lowest push-out bond strength (mean ± SD: 0.41 + 0.4 MPa) [2]. However, the results reported are comparable to the ones obtained by this study. The difference might be due to the fact, that Alnaqbi et al. performed the bond strength testing only on two sections [31].

This study also evaluated the bond strength at three portions of the root with a post at different levels namely, coronal, medium and apical. In the present study, the bond strength was highest in the coronal root level for all tested post systems but did not differ significantly from the other two root levels. These results confirm report by Perdigão. et al. [28] that bonding at the coronal level of the root canal seems to be more reliable than bonding at the apical level. In contrast to our results Kremeier K, et al. [29] for all experimental groups of posts observed superior bond strength in apical compared to coronal sections of the roots.

The study protocol was standardized for all post systems used. Variolink II, a conventional resin cement (Variolink II, Ivoclar Vivadent, Schaan, Lichtenstein) was used for the cementation of all posts. This material has been used in previous studies [18]. Additionally, the literature showed that the type of self-adhesive cement used for the cementation has no influence on bond strength [30].

In this experimental design, mechanical instrumentation, irrigation and filling of root canals with gutta-percha and sealer prior to preparing a post space has been conducted. This approach allowed to provide a clinically realistic situation when bonding posts in endodontically treated teeth as root canal obturation is one of the essential steps [28]. As traces of gutta-percha and sealer filler on dentinal walls of root canal may interfere with bonding in contrast to the clinical situation, the root canals in some studies [29] were not filled with sealer and gutta-percha prior to post space preparation in order to eliminate a possible confounding factor. Future clinical studies should verify the results obtained in our in-vitro study.

## 5. Conclusions

From this study, the following could be concluded:

1. The bond strength of FRC post systems was significantly higher than those of rigid posts of titanium or ZrO_2_.
2. The bond strength was highest in the coronal root level for all tested post systems but did not differ significantly from the other two root levels.
3. Clinicians are advised to use FRC post systems due to their advantageous bond strength and biomimetic mechanical behavior so that root fractures could be avoided, providing that the bond strength did not exceed 10 MPa.

## Figures and Tables

**Table 1:** Post systems used in this study and their chemical compositions.

Post System (Manufacturer)	Chemical Composition	Abbreviation	Post Treatment and Light Curing
Titanium (Mooser) Cendres + Métaux SA, Biel-Bienne, Switzerland	Pure titanium	T	Alloy primer (20 s) and air-thinning No light-curing
Zirconia (Cosmopost) Ivoclar Vivadent AG, Schaan, Liechtenstein	Zirconium oxide (ZrO_2_) ceramic, which consists of ZrO2, HfO_2_, Y_2_O_3_ and Al_2_O_3_.	ZrO	No conditioning No light-curing
Fiber (FRC Postec Plus) Ivoclar Vivadent AG, Schaan, Liechtenstein	Glass fiber, ytterbium Trifluoride, silicon dioxide and dimethacrylate (21%), i.e., Bis-GMA, UDMA and TEGDMA	G	Etching (60 s) using 37% phosphoric acid; water rinsing; air-drying; silane application (60 s) and air-drying. Light-curing
Fiber (Direct) (Everstick post) Stick Tech Ltd., Turku, Finland	Unpolymerized glass fiber E-Glass fibers, methacrylate resin (PMMA, Bis-GMA)	E1	Etching (60 s) using 37% phosphoric acid; water rinsing; air-drying; silane application (60 s) and air-drying. Light-curing
Fiber (indirect) (Everstick post) Stick Tech Ltd., Turku, Finland	Unpolymerized glass fiber E-Glass fibers, methacrylate resin (PMMA, Bis-GMA)	E2	Etching (60 s) using 37% phosphoric acid; water rinsing; air-drying; silane application (60 s) and air-drying. Light-curing
Fiber (PinPost) Dentapreg America Inc., Sarasota, FL, USA	S-glass fibers combined with a blend of methacrylate monomers resin	PP	Etching (60 s) using 37% phosphoric acid; water rinsing; air-drying; silane application (60 s) and air-drying. Light-curing
Injectable Resin/Fiber composite (EverX Posterior) GC Corporation, Tokyo, Japan	Short E-glass fiber filler, barium glass Bis-GMA, PMMA, TEGDMA	LP	No conditioning Light-curing

**Table 2:** The mean bond strength values (mean ± standard deviation (SD)) (in MPa) of T, ZrO, G, E1, E2, PP and LP. Minimum, Maximum and 95%-Confidence Interval of mean bond strength values of T, ZrO, G, E1, E2, PP and LP. Bond strength values with different superscripts a, b, A, B, C are significantly different from each other.

Post Type	Bond Strength (Mean ± SD) [MPa]	*Min-Max (95% CI)* [MPa]
**T**	2.2 ± 1.5 ^a^	0.7–11.2 (1.7–2.7)
**ZrO**	1.9 ± 1.0 ^a,A^	0.2–5.1 (1.4–2.3)
**G**	4.0 ± 1.6 ^b,B^	1.0–11.0 (3.0–4.9)
**E1**	3.6 ± 2.1^b,B^	0.4–8.4 (2.7–4.4)
**E2**	5.3 ± 2.7 ^b,B,C^	1.1–11.1 (4.0–6.7)
**PP**	4.1 ± 2.0 ^b,B^	0.5–9.8 (3.1–5.1)
**LP**	2.6 ± 1.9 ^a^	0.1–9.0 (1.9–3.2)

**Table 3:** The mean bond strength values (mean ± standard deviation (SD)) (in MPa), Minimum, Maximum, and 95%-Confidence Interval of mean bond strength values of all post systems divided by three root levels longitudinally (coronal, middle, and apical).

Root Level	N	Push-Out Bond Strength (Mean ± SD) [MPa]	*Min-Max FS (95% CI)* [MPa]
**Coronal (1st)**	70	3.6 ± 2.2	1.0–11.0 (3.2–4.0)
**Middle (2nd)**	70	3.2 ± 2.1	0.0–11.0 (2.9–3.5)
**Apical (3rd)**	70	3.2 ± 2.3	0.0–10.0 (2.9–3.6)

**Table 4:** Push-out bond strength results (Mean ± SD) MPa and significance values of the mean bond strength values of all post systems T, ZrO, G, E1, E2, PP and LP at all three root levels (coronal (1st), middle (2nd) and apical (3rd) compared to each other. Bold and font values indicate statistical significance (*p* < 0.05).

Post	System	T			ZrO			G			E1			E2			PP			LP		
	Root Level	1st	2nd	3rd	1st	2nd	3rd	1st	2nd	3rd	1st	2nd	3rd	1st	2nd	3rd	1st	2nd	3rd	1st	2nd	3rd
**T**	**1st** (2.0 ± 1.2)	-	1.000	1.000	1.000	1.000	1.000	0.190	**0.005**	0.190	**0.009**	0.975	0.325	**0.000**	**0.000**	**0.000**	**0.037**	0.624	**0.000**	0.080	1.000	1.000
	**2nd** (2.7 ± 2.1)	1.000	-	1.000	1.000	0.985	0.998	0.961	0.275	0.961	0.380	1.000	0.991	**0.001**	**0.001**	**0.001**	0.685	1.000	**0.048**	0.841	1.000	0.841
	**3rd** (2.0 ± 1.0)	1.000	1.000	-	1.000	1.000	1.000	0.190	**0.005**	0.190	**0.009**	0.975	0.325	**0.000**	**0.000**	**0.000**	**0.037**	0.624	**0.000**	0.080	1.000	1.000
**ZrO**	**1st** (2.0 ± 1.1)	1.000	1.000	1.000	-	1.000	1.000	0.230	**0.007**	0.230	**0.012**	0.985	0.380	**0.000**	**0.000**	**0.000**	**0.048**	0.685	**0.000**	0.100	1.000	1.000
	**2nd** (1.7 ± 1.0)	1.000	0.985	1.000	1.000	-	1.000	0.062	**0.001**	0.062	**0.002**	0.841	0.125	**0.000**	**0.000**	**0.000**	**0.009**	0.325	**0.000**	**0.022**	1.000	1.000
	**3rd** (1.9 ± 1.2)	1.000	0.998	1.000	1.000	1.000	-	0.125	**0.002**	0.125	**0.005**	0.941	0.230	**0.000**	**0.000**	**0.000**	**0.022**	0.499	**0.000**	**0.048**	1.000	1.000
**G**	**1st** (3.7 ± 1.9)	0.190	0.961	0.190	0.230	0.062	0.125	-	1.000	1.000	1.000	0.999	1.000	0.380	0.438	0.438	1.000	1.000	0.975	1.000	0.325	**0.012**
	**2nd** (4.4 ± 1.4)	**0.005**	0.275	**0.005**	**0.007**	**0.001**	**0.002**	1.000	-	1.000	1.000	0.624	0.999	0.985	0.991	0.991	1.000	0.975	1.000	1.000	**0.012**	**0.000**
	**3rd** (3.7 ± 1.4)	0.190	0.961	0.190	0.230	0.062	0.125	1.000	1.000	-	1.000	0.999	1.000	0.380	0.438	0.438	1.000	1.000	0.975	1.000	0.325	**0.012**
**E1**	**1st** (4.2 ± 2.3)	**0.009**	0.380	**0.009**	**0.012**	**0.002**	**0.005**	1.000	1.000	1.000	-	0.742	1.000	0.961	0.975	0.975	1.000	0.991	1.000	1.000	**0.022**	**0.000**
	**2nd** (3.0 ± 1.9)	0.975	1.000	0.975	0.985	0.841	0.941	0.999	0.624	0.999	0.742	-	1.000	**0.007**	**0.009**	**0.009**	0.941	1.000	0.190	0.985	0.995	0.4999
	**3rd** (3.6 ± 2.2)	0.325	0.991	0.325	0.380	0.125	0.230	1.000	0.999	1.000	1.000	1.000	-	0.230	0.275	0.275	1.000	1.000	0.914	1.000	0.499	**0.028**
**E2**	**1st** (5.3 ± 2.8)	**0.000**	**0.001**	**0.000**	**0.000**	**0.000**	**0.000**	0.380	0.985	0.380	0.961	**0.007**	0.230	-	1.000	1.000	0.795	0.080	1.000	0.624	**0.000**	**0.000**
	**2nd** (5.3 ± 2.9)	**0.000**	**0.001**	**0.000**	**0.000**	**0.000**	**0.000**	0.438	0.991	0.438	0.975	**0.009**	0.275	1.000	-	1.000	0.841	0.100	1.000	0.685	**0.000**	**0.000**
	**3rd** (5.3 ± 2.7)	**0.000**	**0.001**	**0.000**	**0.000**	**0.000**	**0.000**	0.438	0.991	0.438	0.975	**0.009**	0.275	1.000	1.000	-	0.841	0.100	1.000	0.685	**0.000**	**0.000**
**PP**	**1st** (4.0 ± 1.9)	**0.037**	0.685	**0.037**	**0.048**	**0.009**	**0.022**	1.000	1.000	1.000	1.000	0.941	1.000	0.795	0.841	0.841	-	1.000	1.000	1.000	0.080	**0.001**
	**2nd** (3.4 ± 1.4)	0.624	1.000	0.624	0.685	0.325	0.499	1.000	0.975	1.000	0.991	1.000	1.000	0.080	0.100	0.100	1.000	-	0.685	1.000	0.795	0.100
	**3rd** (4.7 ± 2.6)	**0.000**	**0.048**	**0.000**	**0.000**	**0.000**	**0.000**	0.975	1.000	0.975	1.000	0.190	0.914	1.000	1.000	1.000	1.000	0.685	-	0.998	**0.001**	**0.000**
**LP**	**1st** (3.9 ± 2.1)	0.080	0.841	0.080	0.100	**0.022**	**0.048**	1.000	1.000	1.000	1.000	0.985	1.000	0.624	0.685	0.685	1.000	1.000	0.998	-	0.155	**0.003**
	**2nd** (2.1 ± 1.4)	1.000	1.000	1.000	1.000	1.000	1.000	0.325	**0.012**	0.325	**0.022**	0.995	0.499	**0.000**	**0.000**	**0.000**	0.080	0.795	**0.001**	0.155	-	1.000
	**3rd** (1.5 ± 1.0)	1.000	0.841	1.000	1.000	1.000	1.000	**0.012**	**0.000**	**0.012**	**0.000**	0.499	**0.028**	**0.000**	**0.000**	**0.000**	**0.001**	0.100	**0.000**	**0.003**	1.000	-

## Data Availability

No data reported.
